# Arquivos Brasileiros de Cardiologia (ABC Cardiol) and the new classification Qualis of Coordenação de Aperfeiçoamento de Pessoal de Nível Superior (CAPES)

**DOI:** 10.5935/abc.20190206

**Published:** 2019-09

**Authors:** Carlos E. Rochitte, Alexandre Schaan de Quadros, Amanda Guerra de Moraes Rego Sousa, Ana Marice Teixeira Ladeia, Andréa Araujo Brandão, Andrea De Lorenzo, Andrei Sposito, Antonio Luiz Pinho Ribeiro, Claudio Tinoco Mesquita, Fernanda Marciano Consolim Colombo, José Antônio Marin-Neto, Ludhmila Abrahão Hajjar, Marcelo Bertolami, Ricardo Stein, Sandra Fuchs, Sergio Emanuel Kaiser, Romeu Sergio Meneghelo, Gláucia Maria Moraes de Oliveira

**Affiliations:** 1 Instituto do Coração (Incor), São Paulo, SP - Brazil; 2 Programa de Pós-Graduação do Instituto de Cardiologia do Rio Grande do Sul/Fundação Universitária de Cardiologia (IC/FUC), Porto Alegre, RS - Brazil; 3 Programa de Pós-graduação de Medicina/Tecnologia e Intervenção em Cardiologia do Instituto Dante Pazzanese, São Paulo, SP - Brazil; 4 Programa de Pós-graduação em Medicina e Saúde Humana da Escola Bahiana de Medicina e Saúde Pública, Salvador, BA - Brazil; 5 Programa de Pós-Graduação em Ciências Médicas Universidade do Estado do Rio de Janeiro (UERJ), Rio de Janeiro, RJ - Brazil; 6 Programa de Pós-Graduação em Ciências Cardiovasculares do Instituto Nacional de Cardiologia (INC), Rio de Janeiro, RJ - Brazil; 7 Faculdade de Ciências Médicas, UNICAMP, Campinas, SP - Brazil; 8 Programa de Pós-Graduação em Ciências da Saúde - Infectologia e Medicina Tropical da Universidade Federal de Minas Gerais, Belo Horizonte, MG - Brazil; 9 Programa de Pós-Graduação em Ciências Cardiovasculares da Universidade Federal Fluminense (UFF), Rio de Janeiro, RJ - Brazil; 10 Programa de Pós-Graduação em Medicina da Universidade Nove de Julho (UNINOVE), São Paulo, SP - Brazil; 11 Unidade de Cardiologia Intervencionista da Divisão de Cardiologia da Faculdade de Medicina de Ribeirão Preto da Universidade de São Paulo, São Paulo, SP - Brazil; 12 Programa de Pós-Graduação em Cardiologia da Universidade de São Paulo (USP), São Paulo, SP - Brazil; 13 Programa de Pós-graduação (Doutorado) do Instituto Dante Pazzanese de Cardiologia, São Paulo, SP - Brazil; 14 Programa de Pós-Graduação em Cardiologia e Ciências Cardiovasculares, Universidade Federal do Rio Grande do Sul, Hospital de Clínicas de Porto Alegre, Porto Alegre, RS - Brazil; 15 Programa de Pós-Graduação em Cardiologia e Ciências Cardiovasculares da Faculdade de Medicina da UFRGS, Porto Alegre, RS - Brazil; 16 Programa de Pós-Graduação em Fisiopatologia Clínica e Experimental da Universidade Estadual do Rio de Janeiro (UERJ), Rio de Janeiro, RJ - Brazil; 17 Programa de Mestrado Profissional Associado à Residência em Medicina Cardiovascular do Instituto Dante Pazzanese de Cardiologia, São Paulo, SP - Brazil; 18 Programa de Pós-Graduação em Cardiologia da Universidade Federal do Rio de Janeiro (UFRJ), Rio de Janeiro, RJ - Brazil

**Keywords:** Education, Medical, Graduate/trends, Program Evaluation, Periodicals as Topic, Research Policy Evaluation, Journal Impact Factor

The Arquivos Brasileiros de Cardiologia (ABC), the official scientific journal of the Brazilian Society of Cardiology (BSC), is currently the journal in Cardiology with the highest impact in Brazil and Latin America, with a 2018 JCR impact factor of 1.679.^1^ During its history, the ABC has played an important role of publishing scientific papers produced in Brazil in journals indexed in the most prominent English-language, international databases. It thus acts as a window to the world of the Brazilian scientific production.

The mission of the ABC is to disseminate the results of national and international studies on cardiovascular diseases, to foster scientific debate on the area by means of review articles, viewpoints, editorials, letters and others, and to publish BSC scientific guidelines and standards. The journal’s commitment is in line with the mission of the BSC to expand and spread the knowledge about cardiovascular science, to represent and promote the development of cardiologists and to take actions for the cardiovascular health in Brazil.

The ABC have their own regulations. The Editor-in-Chief is elected for a four-year-term, and mandate’s renewal is subjected to the approval by a judging committee composed by members of the Council and published by public notice. The journal has 12 associate editors, eight of them professors of postgraduate programs, working in the respective areas: Medical Cardiology; Surgical Cardiology; Interventional Cardiology; Pediatric Cardiology/Congenital Cardiopathy; Arrythmia/Pacemaker/Noninvasive diagnostic techniques; Basic or Experimental Research; Epidemiology/Statistics; Arterial Hypertension; Ergometry; Exercise and Cardiac Rehabilitation. The editors are chosen by the Editor-in-Chief. The editorial board, composed of approximately 100 members, undergoes a complete revision every four years, considering qualitative prerequisites, such as scientific production and academic activity.

The number of articles recently published in the ABC reflects the consistency of the editorial line of the journal, with 185, 242 and 187 articles published in 2017, 2018 and in the current year, respectively. The growing interest in publishing in the ABC is clearly shown by the increasing number of articles submitted to the journal - 650 in 2017, 771 in 2018 and 734 so far in 2019. The current acceptance rate is lower than 20% of the articles submitted and approximately 30% of the articles rejected are transferred to other journals of the ABC group, the IJCS and the ABC *imagem cardiovascular*, contributing to the scientific quality of these Brazilian journal partners.

The number of original articles published in the ABC in 2017, 2018 and 2019, respectively, was 96 (65 of studies conducted in postgraduate programs), 98 (53 of postgraduate programs) and 40 (32 of postgraduate programs). Therefore, nearly 65% of the original articles published in the ABC are contributions from postgraduate programs. These data show that the ABC meet the strict high-quality requirements of *the Coordenação de Aperfeiçoamento de Pessoal de Nível Superior* (CAPES, Coordination for the Improvement of Higher Education Personnel) regarding the quality of intellectual production of the Cardiology postgraduate programs, including articles published in scientific journals. To meet the criteria imposed by CAPES is a challenging task that requires the combined efforts of postgraduate programs and medical societies, aiming at making the scientific production from postgraduate programs of all academic institutions available and renown by national and international peers.

The ABC have followed an international trend of open science to give value to publications that make scientific data universally accessible. As an example, the European Community has the objective to make all the scientific production universally available by the year of 2020, similar to large research funding agencies, such as the National Institute of Health (NIH), which already requires that all scientific data produced by projects funded by the agency are openly published.

We should support national scientific journals, with the aim of improving their impact factor and their position in the global science scenario. Also, publication of national studies on cardiovascular disease should be encouraged, as they represent the main cause of mortality in Brazil and in the world. One of the aspects considered important by CAPES is the social insertion of the postgraduate programs, seeking to improve life conditions of the population. However, studies on Brazilian populations, with their particular socioeconomic characteristics, attract little or no attention from the international scientific community. These studies would then be benefited from the promotion by CAPES’ evaluation system; this would construct a strong national exchange network. This social role has been played by the ABC. In this regard, it would be interesting that CAPES, by means of Qualis, evaluated the ABC, which is the main journal on the fight against cardiovascular disease, allowing the sharing of successful experiences in this sense.

Finally, the ABC are truly a “world-class” journal, with 70 years of history, high internationalization, with continuous support from the BSC, and composed mostly of articles produced in postgraduate programs. The ABC are the most important journal on Cardiology and Cardiovascular Sciences, with the highest impact factor in Brazil and Latin America. It is undoubtedly the ideal journal to publish scientific production of postgraduate programs.

The ABC is currently classified as B1 category, which discourages the submission by authors of postgraduate programs to this journal, which is based in Brazil, has renown impact factor and is highly internationalized, when compared to other national and international journals of internal medicine, with comparable impact factors, and classified in higher levels by CAPES.

Therefore, the difficulty imposed by CAPES classification restricts the use of national and specialized journals as the ABC. We believe that the A2 level encompasses journals of similar scientific reputation to the ABC and would be the most suitable to this journal.

Finally, it is the opinion of the authors of this editorial and large part of the brazilian postgraduate programs in Cardiology, that we respectfully ask the revision of the latest classification of the ABC given by CAPES, based on the potential direct benefits to the national scientific community and to the postgraduate programs in Brazil.
